# Chromatin Remodeling via Retinoic Acid Action during Murine Spermatogonial Development

**DOI:** 10.3390/life13030690

**Published:** 2023-03-03

**Authors:** Christine Schleif, Rachel Gewiss, Michael Griswold

**Affiliations:** Center for Reproductive Biology, School of Molecular Biosciences, Washington State University, Pullman, WA 99164, USA

**Keywords:** chromatin, retinoic acid, testis, spermatogenesis, ATAC-seq

## Abstract

Spermatogonial differentiation is a process that commits germ cells to the complex process of spermatogenesis. Spermatogonial differentiation is mediated by the action of retinoic acid, which triggers major morphological and transcriptional changes. While these transcriptional changes have been well explored, there has been little effort devoted to epigenetic regulation surrounding spermatogonial development. This study aimed to uncover the timing and dynamics of chromatin organization during spermatogonial development within the context of these transcriptional changes. Using germ cell synchrony and the assay for transposase accessible chromatin and next generation sequencing (ATAC-seq) to isolate subpopulations of developing spermatogonia and identify accessible regions within their genome, we found that 50% of accessible regions in undifferentiated spermatogonia were condensed following retinoic acid action within 18 h. Surprisingly, genes with known functional relevance during spermatogonial development were accessible at all times, indicating that chromatin state does not impact transcription at these sites. While there was an overall decrease in gene accessibility during spermatogonial development, we found that transcriptionally active regions were not predictive of chromatin state.

## 1. Introduction

Spermatogenesis is a complex process whereby relatively undifferentiated germ cells undergo morphological, transcriptional, and epigenetic changes as they mature into sperm. These critical changes not only underlie the functionality of cells in each stage of development but also allow for the identification of cell types based on these characteristics. Gross morphological traits can be used to identify the four main types of male germ cells: spermatogonia, spermatocytes, round spermatids, and elongating spermatids [1]. The association of these different cell types in the adult testis generates the cycle of the seminiferous epithelium, where 12 stages are defined in the mouse based on cell associations present in a cross-section of a seminiferous tubule [2,3]. Such morphological traits are useful, especially when identifying cell types histologically in testis cross-sections or when only the broad cell type categorization is needed. Spermatid classification can be further assessed based on acrosome shape and position [4]. However, fewer obvious morphological differences exist to distinguish the different subtypes of spermatogonia and spermatocytes.

Multiple studies have attempted to uncover the transcriptional changes during male germ cell development [5,6,7,8]. Several cell populations have key regulators which display dramatic changes in protein levels. One such example is stimulated by retinoic acid gene 8 (STRA8), which is highly expressed in response to retinoic acid (RA) following the A-to-A1 transition of spermatogonial differentiation [9,10]. KIT is another well-used marker of differentiating cells and clearly represents the switch between undifferentiated A spermatogonia (A_undiff_) and differentiating A1 spermatogonia (the transition between these two cell types is termed the A-to-A1 transition) [11,12]. While the A-to-A1 transition and meiotic stages have useful markers for some subtypes of these cells, spermatogonial subtypes are particularly difficult to distinguish. By synchronizing spermatogenesis in the mouse, we have recently described transcriptional changes that occur in each subtype of differentiating spermatogonia [5]. When taken together, these transcriptional changes can be useful to distinguish differentiating spermatogonial subtypes from each other and can be used to assign more specific identities to data clusters in single-cell sequencing datasets [7,8].

Previous studies have examined chromatin state and possible chromatin modifiers in germ cells using the assay for transposase-accessible chromatin with sequencing (ATAC-seq) and chromatin immunoprecipitation with sequencing (ChIP-seq) [13,14]. However, only the spermatogonial stem cell population and later stages of spermatogenesis, such as pachytene spermatocytes, and spermatids were examined [13,14]. The spermatogonial population has been heretofore unexplored beyond broad transmission electron microscopy (TEM) studies showing that chromatin condensation gradually increases during spermatogonial development [15,16]. Thus, the genomic locations of chromatin condensation and any possible effects of this on transcription in spermatogonia remain unknown.

In this study, we characterized where in the genome chromatin compaction was occurring throughout spermatogonial development. Further, we correlated these changes in chromatin compaction with RNA-seq data to determine whether these changes were broad throughout the genome, or if they correlated with transcriptome changes seen during spermatogonial development. We synchronized mouse spermatogenesis to isolate purified spermatogonial subpopulations at several points during spermatogonial differentiation and then performed ATAC-seq to characterize the accessibility of chromatin in each of the spermatogonial subpopulations.

## 2. Materials and Methods

### 2.1. Animals

All animal experiments were approved by the Washington State University Animal Care and Use Committees and were conducted in accordance with the principles for the care and use of research animals of the National Institutes of Health. Tg(Piwil2/EGFP)1GHan/J mice (denoted here as PIWIL2-eGFP) were obtained from the Jackson Laboratories (stock no. 012276) [17]. These mice express GFP under control of the piwi-like RNA mediated gene silencing 2 (*Piwil2*) promoter, and expression is seen prenatally through early spermatids; these mice were produced from a C57BL/6 x SJL background [18]. Animals were housed in a humidity- and temperature-controlled environment with food and water provided ad libitum. At the time of tissue collection, mice were euthanized via carbon dioxide asphyxiation followed by either decapitation (less than 21 dpp) or cervical dislocation (21 dpp and older). Testes from one mouse were pooled and used for each sample, with two biological replicates per timepoint.

### 2.2. Genotyping

Tail clips were taken from neonatal mice to identify genotypes prior to euthanasia. These tail clips were lysed in 75 µL of 25 mM NaOH, 200 µM EDTA (pH 12) in a 95 °C heat block for 1 h, with manual dissociation at 30 min. A total of 75 µL of neutralization buffer (40 mM Tris-HCl, pH 5) was then added, before DNA was used for genotyping reactions. Mice were assayed for the presence of the PIWIL2-eGFP transgene using primers specific to eGFP (forward: 5′-CTG ACT CCT GAT GAA GTG TTA TAG CC-3′; reverse: 5′-TCC TTG AAG AAG ATG GTG CGC TCC T-3′). The presence of a band at ~500 bp signified the presence of the transgene.

### 2.3. WIN 18,446/RA Treatments

Spermatogenesis synchronization was performed as previously described [5,19]. Briefly, mice were pipette-fed with 100 mg/kg body weight WIN 18,446 suspended in 1% gum tragacanth every 24 h for 7 days starting at 2 dpp. At 9 dpp, mice were injected intraperitoneally with 200 µg RA diluted in 10 µL dimethyl sulfoxide (DMSO). Mice were then euthanized, and testes were collected at 18, 48, or 120 h following the RA injection. For “0 h” collections, mice received 7 days of WIN 18,446 treatment and were euthanized without receiving an RA injection.

### 2.4. Cell Sorting

Testes to be used for cell sorting were collected and the tunica removed. Tubules were placed in 1X Hank’s Balanced Salt Solution (HBSS, 14175145, ThermoFisher, Waltham, MA, USA) until transfer to a Petri dish with 5 mL trypsin/EDTA (25200072, ThermoFisher, Waltham, MA, USA), and 0.5 mL DNase (7 mg/mL in HBSS, 9003-98-9, Sigma, San Jose, CA, USA). Tubules were manually dissociated via pipetting and then incubated for 5 min at 37 °C. A total of 1 mL of DNase was then added to the dish, and tubules were further dissociated. The cells were incubated for another 5 min at 37 °C, and these steps were repeated twice more. A total of 850 µL of fetal bovine serum (FBS) was then added to inactivate the trypsin, and the suspension was strained through a 30 µm filter. The Petri dish was rinsed with 4 mL of HBSS to remove any remaining cells which were then also filtered, followed by a final 1 mL HBSS wash through the filter. The cell number in the suspension was estimated using a hemocytometer. Cells were spun down at 600× *g* for 7 min at 4 °C, then resuspended in 12.5% DNase solution diluted in Dulbecco’s phosphate-buffered saline (DPBS) (14190235, ThermoFisher, Waltham, MA, USA) at 4 million cells/mL. Fluorescence-activated cell sorting (FACS) was performed with an SH800 machine (Sony Biotechnology, San Jose, CA, USA) to distinguish eGFP-positive and eGFP-negative populations. eGFP-positive cells were collected into a tube containing 0.5 mL DPBS. As previously described in Gewiss et al. (2021), eGFP-positive cells were collected at a rate >95% [5].

### 2.5. ATAC-Seq Preparation

A modified version of the Omni-ATAC protocol was used to generate ATAC-seq libraries [20,21]. After cells were collected from FACS, 50,000 cells per sample were spun down at 500× *g* for 7 min at 4 °C. Cells were then resuspended in 500 µL cold solution comprised of 50% FBS, 40% DPBS, 10% DMSO. Cells were cryopreserved at -80 °C overnight. Prior to the ATAC-seq assay, cells were thawed in a 37 °C water bath, pelleted, washed with cold PBS, and tagmented as described in Buenrostro et al. (2013) with modifications [20,21]. Briefly, cell pellets were resuspended in lysis buffer, pelleted, and tagmented using the enzyme and buffer provided in the Nextera Library Prep Kit (Illumina, San Diego, CA, USA). Tagmented DNA was then purified using the MinElute PCR purification kit (Qiagen, Hilden, Germany), amplified with 10 cycles of PCR, and purified using Agencourt AMPure SPRI beads (Beckman Coulter, Brea, CA, USA). The resulting material was quantified using the KAPA Library Quantification Kit for Illumina platforms (KAPA Biosystems, Wilmington, MA, USA) and sequenced with PE42 sequencing on the NextSeq 500 sequencer (Illumina, San Diego, CA, USA).

### 2.6. Data Processing and Analysis

ATAC-seq analysis used the mouse mm10 genome and read alignment was performed using the BWA algorithm (mem mode, default settings) [22]. Duplicate reads were removed and unique reads with matched pairs were used for further analysis. Alignments were extended in silico at their 3′-ends to a length of 200 bp and assigned to 32-nt bins along the genome. The resulting histograms (genomic “signal maps”) were stored in bigWig files. Peaks were identified using the MACS 2.1.0 algorithm at a cutoff of *p*-value 1 × 10^−7^, without a control file, and with the –nomodel option. Peaks that were on the ENCODE blacklist of known false ChIP-Seq peaks were removed. Signal maps and peak locations were used as input data to Active Motif’s (Carlsbad, CA, USA) proprietary analysis program, which creates Excel tables containing detailed information on sample comparison, peak metrics, peak locations, and gene annotations. Sequencing metrics are provided in the Supplementary Materials. For differential analysis, reads were counted in all merged peak regions (using Subread v1.5.2, accessed 16 December 2021), and the replicates for each condition were compared using DESeq2 (v1.24.0, accessed 16 December 2021) [23]. Peak tracks were generated with EaSeq v1.111 [24]. Heatmap creation was performed using R v4.2.1 [25] with packages ggplot2 [26], ggdendro [27], grid [25], and tidyr (all accessed 16 December 2021) [28]. Genes which had transcript values of 0 for all timepoints were omitted to allow better clustering. Genes names, peak values, and z-scores for heatmaps are given in the Supplementary Materials.

## 3. Results

We isolated spermatogonial subpopulations with FACS using a PIWIL2-eGFP mouse line. The PIWIL2-eGFP transgene is expressed prenatally in all germ cells and in adults through early spermatids, which allowed us to use fluorescence to isolate germ cells from other testicular cell types. Further, we used a previously characterized WIN 18,446/RA spermatogenesis synchrony protocol to produce testes highly enriched for given types of germ cells [19]. We have previously found that the timing of spermatogonial development in synchronized and RA treated mice is relatively the same as seen in vivo [5,16].

Additionally, this synchrony remains tightly coordinated for over the six weeks following RA administration [19]. This ensured that cell populations were highly pure as only spermatogonia were present in the 120 h following RA administration [5,16].

We collected two ATAC-seq replicates following synchronization at 0, 18, 48, and 120 h post-RA, which correspond to the A_undiff_, A1/A2, A3, and B spermatogonial populations, respectively. These timepoints allowed us to see the impact on the chromatin state of RA-induced spermatogonial differentiation. The 0 h samples represent the A_undiff_ spermatogonial population prior to RA action. Previous transcript analysis has shown that for many transiently induced RA-influenced genes, transcript levels increase through 18 h post-RA and then return to near-basal levels by 48 h [5,29,30]. The 120 h timepoint allowed us to assess the chromatin changes that occurred over the full course of spermatogonial development. Principal component analysis (PCA) revealed a good replicability between the two biological replicates samples collected for each timepoint (Figure S1).

**Figure 1:**
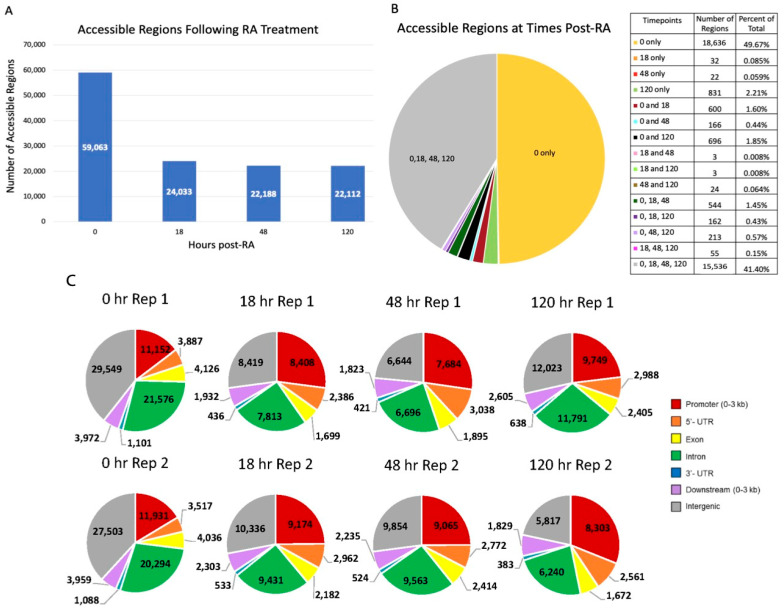
Accessible regions. (**A**) The shared accessible regions between the two replicates at each timepoint for 0, 18, 48, and 120 h post-RA revealed a monotonic decrease in accessibility following RA treatment. (**B**) Unique accessible regions for each comparison of times post-RA treatment. (**C**) Accessible regions grouped by genomic traits for each replicate and timepoint. Notably, the total regions here may show a greater total than Figure 1A, as these represent regions called in a single replicate not only those which were accessible in both replicates of a timepoint.

We first analyzed the number of ATAC-seq peaks in each sample, representing the areas of open chromatin. Figure 1A details the number of genomic regions considered accessible in both biological replicates, which were then used for further analysis. Shared accessible regions between timepoints are shown in Figure 1B. The greatest number of accessible regions were found exclusively at the 0 h timepoint (18,636 regions), followed by those regions which were accessible at all assayed timepoints (15,536 regions).

**Figure 2:**
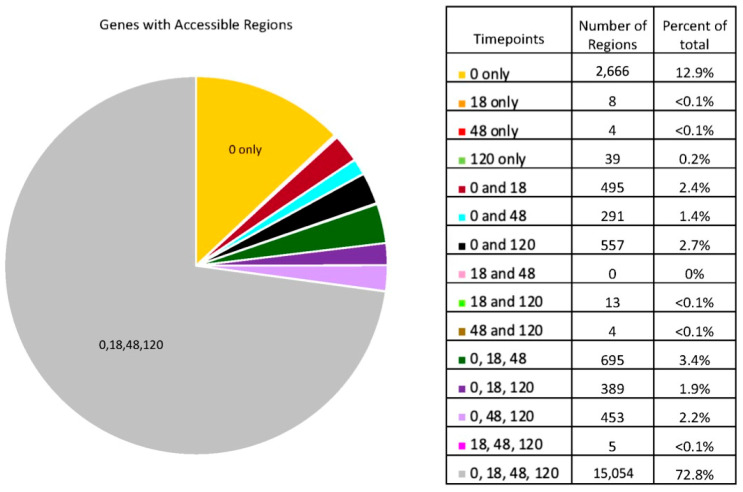
Accessible genes. Genes with associated accessible regions at 0, 18, 48, and/or 120 h post-RA showed that 72.8% of accessible genes were accessible at all timepoints. The numbers in the table show the number of regions per category, and percentages of the total are shown to the right of the legend. Genes with accessible regions were included for a timepoint if both biological replicates indicated that the gene contained and/or was within 10 kb of an accessible region.

A breakdown of the genomic location of these peaks is shown in Figure 1C. The majority of these peaks were either in an intergenic region, in introns, or in the promoter region. In order to gain a better perspective on how these accessible regions may affect transcription, we determined which accessible regions were near (within 10 kb of) an annotated gene (Figure S2). At all assayed timepoints, accessible regions near genes comprised ~68–83% of the total number of accessible regions. Of these accessible regions near genes, we then compared the timepoints to see how the chromatin was changing over time. A breakdown of the 20,673 genes with associated accessible chromatin is shown in Figure 2. There were 15,054 genes with accessible regions at all four timepoints (only counted if both biological replicates for a timepoint indicated accessibility). Genes with associated peaks at a single timepoint or shared between only two or three timepoints were minimal, with the exception of 2666 genes with accessible regions only in the A_undiff_ population.

Figure S3 gives the peak distribution over the gene bodies for these accessible genes. The resulting accessibility is enriched at the transcription start sites (TSS) of genes at all timepoints.

**Figure 3:**
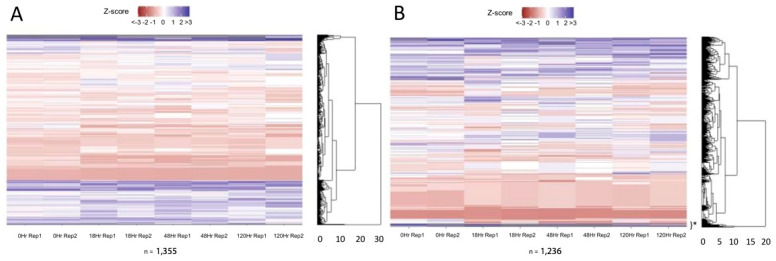
Peak variance relative to transcript regulation. Number of peaks variance shown relative to the mean for genes with significantly (**A**) decreased or (**B**) increased transcript counts by 120 h post-RA relative to 0 h post-RA. List of genes’ number of peaks per timepoint and the plotted z-Scheme 5 (Figure 4 and Figure S5). The two most common categories for chromatin accessibility were genes accessible at all timepoints, or those only accessible at 0 h post-RA (Figure 2). For those genes only accessible at 0 h, there was very little variation in transcription across spermatogonial development (Figure 4A). The same trend was seen for those genes which were accessible at all timepoints (Figure 4B). Thus, those genes which were accessible only prior to RA administration and those accessible at all timepoints showed similar transcript expression patterns.

To characterize global trends for peaks around genes, we generated heatmaps for genes which are either down- or upregulated by 120 h post-RA administration [5] (Figure 3 and Figure S4). For both the downregulated and upregulated genes, the majority have a similar number of peaks at all timepoints.

We next examined some classical marker transcripts for different spermatogonial developmental stages. Prior to the A-to-A1 transition, A_undiff_ spermatogonia display relatively high transcript levels of characteristic markers, including neurogenin 3 (*Neurog3*), SRY-box transcription factor 3 (*Sox3*), zinc finger and BTB domain containing 16 (*Zbtb16*), lin-28 homolog A (*Lin28a*), nanos C2HC-type zinc finger 2 (*Nanos2*), and POU class 5 homeobox 1 (*Pou5f1*, formerly *Oct3/4*) [5,31,32,33,34,35,36,37]. These genes then have decreased transcript levels following RA action and remain at low levels throughout spermatogonial development [5]. Interestingly, all of these genes contained regions of accessible chromatin at all of the assayed timepoints (Figure 5). *Pou5f1* and *Zbtb16* showed fewer peaks in timepoints following RA-triggered differentiation. Several of these genes also showed decreases in peak amplitude, seen notably in Peak 1 of *Neurog3* and Peak 1 of *Sox3*.

We next looked at additional genes with high expression in A_undiff_ spermatogonia and significantly decreased expression following RA action that were previously identified as potential novel A_undiff_ markers [5]. Five genes (Enc1, Onecut2, Ptch1, Sdc4, and Zfp462) fitting this description were selected based on their expression pattern and analyzed for accessibility (Figure 6A–E). Little is known about these genes’ functions in spermatogonia. Interestingly, these differentially expressed genes (DEGs) with high transcript expression in the A_undiff_ population were not exclusively accessible at 0 h but rather showed some accessibility at all timepoints. However, all these genes displayed additional examples of a reduction in accessibility, seen clearly as a reduction in peak count following RA action in Enc1 peaks 1, 3, and 5 (Figure 6A).

Additionally, we examined genes known to have increased transcript levels following the A-to-A1 transition. Potential targets which increase expression following RA action include those stimulated by retinoic acid gene 8 (*Stra8*) and the KIT proto-oncogene, receptor tyrosine kinase (*Kit*) [5,11,12,38,39,40,41]. Our data indicate that there are accessible chromatin regions in and near *Stra8*, even in A_undiff_ spermatogonia, when transcript levels are minimal (Figure 7A). Further, *Kit* has accessible chromatin in/near the gene body in both A_undiff_ spermatogonia at 0 h post- RA and in differentiating spermatogonia post-RA injection (Figure 7B).

As previously discussed, the majority of accessible genes were accessible at all timepoints. We thus took a particular interest in the subset of genes with accessible regions only in undifferentiated spermatogonia (0 h), which made up ~13% of the total number of genes. For these 2666 genes exclusively accessible in undifferentiated spermatogonia, we looked for correlations in transcription from our previous dataset. If chromatin compaction was affecting the transcription of these genes, there should be fewer transcripts at assayed timepoints following RA action. We cross-referenced these genes with genes showing significantly lower transcript levels following RA treatment from our previously published data, resulting in 111 genes of interest [5]. These 111 genes did not show a testicular or spermatogenic link when run through gene ontology (Figure S6).

## 4. Discussion

Our results support the general knowledge that has been ascertained via TEM—that there is a relatively large amount of accessible chromatin prior to the A-to-A1 transition of spermatogonial differentiation, and this amount declines as spermatogonial development progresses [15,16]. Our data revealed that approximately half the number of accessible regions present at 0 h remained following the A-to-A1 transition. The number of accessible regions 18 h post-RA then remained relatively constant at 48 and 120 h post-RA (Figure 1A). It is well-established that RA action at the A-to-A1 transition causes drastic physiological changes leading to the commitment to meiosis [42]; thus, our data indicate that RA is responsible for triggering a dramatic decrease in chromatin accessibility as well.

While chromatin compaction increased overall during the time of spermatogonial development, we assessed how this may impact transcriptional regulation. Globally, we examined genes which are known to possess significantly decreased (Figure 3A) or increased (Figure 3B) transcript levels by 120 h post-RA relative to the A_undiff_ population. Genes from both the down- and upregulated categories showed relatively little change in the number of peaks called during the time course. Interestingly, there was a small subsection of upregulated genes which showed a greater number of peaks prior to RA administration, then showed a decline in peak number following RA action (Figure 3B, denoted with *). The presence of this cluster, along with the relatively unchanged number of peaks for other upregulated genes, indicates an inability to predict chromatin accessibility from transcriptional patterns.

**Figure 4:**
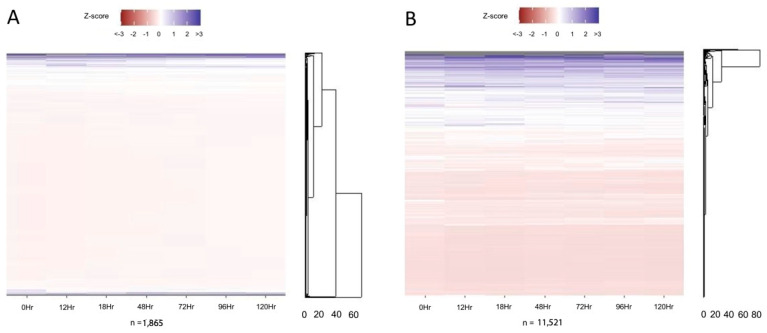
Accessible gene and transcript values. Heatmaps of transcript values during spermatogonial development for genes which have accessible chromatin at (**A**) 0 h only or (**B**) 0, 18, 48, and 120 h (all times). Transcript counts and plotted z-scores are provided in the Supplementary Materials. Transcript data from [5].

Conversely, we examined transcriptional patterns for genes with characterized accessibility patterns, focusing on those genes accessible at 0 h only or accessible at all times as these represented the majority of genes with accessible regions (Figure 4). These genes showed very limited changes in transcription throughout the course of spermatogonial development. Thus, it appears that on a global scale, accessibility is unable to predict transcription patterns in spermatogonia.

**Figure 5:**
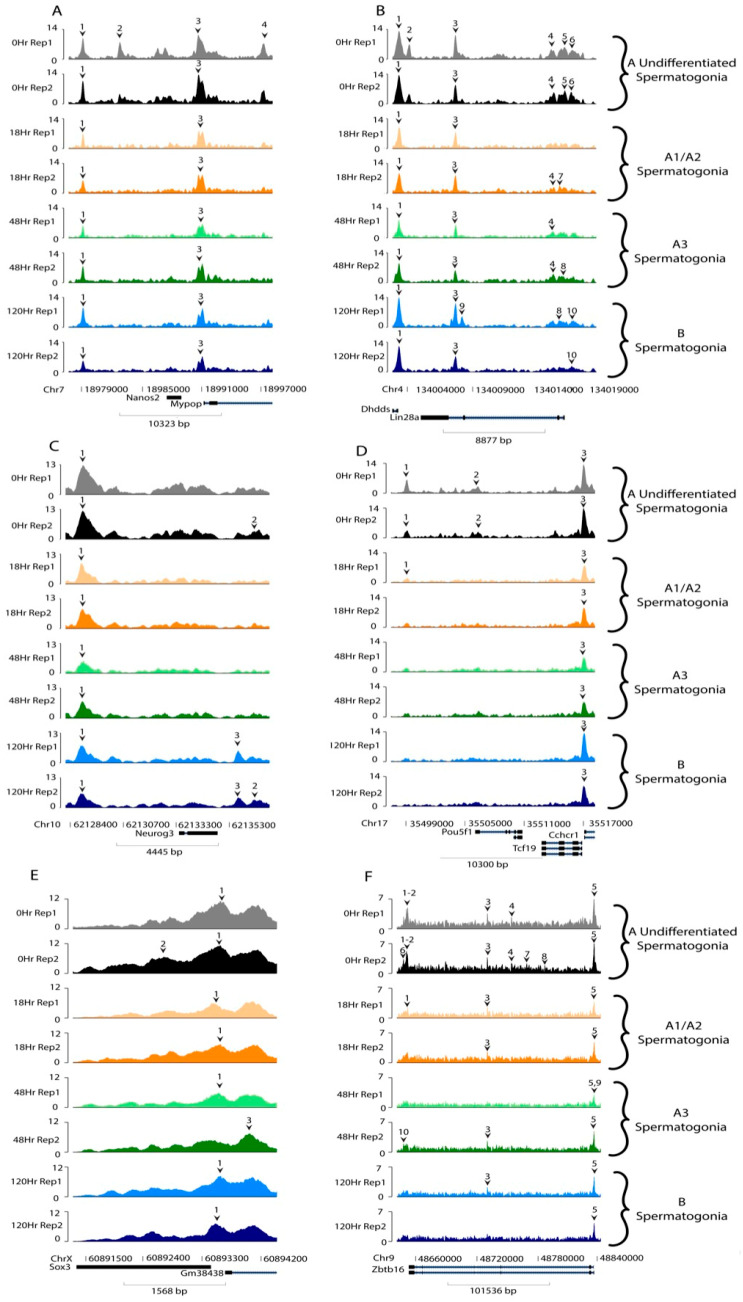
Accessibility of classical markers. Relative sequencing tracks the mapping accessibility of common spermatogonial genes and immunohistochemical markers. Numbered carats denote significant accessibility peaks for each gene. Data shown for both replicates of each timepoint: 0 h (yellow and orange), 48 h (light and dark green), and 120 h (light and dark blue). The genes represented here include (**A**) Nanos2, (**B**) Lin28a, (**C**) Neurog3, (**D**) Pou5f1, (**E**) Sox3, and (**F**) Zbtb16, all of which show a decreasing amplitude and width of peaks between the undifferentiated and differentiating spermatogonia corresponding with decreasing accessibility.

**Figure 6:**
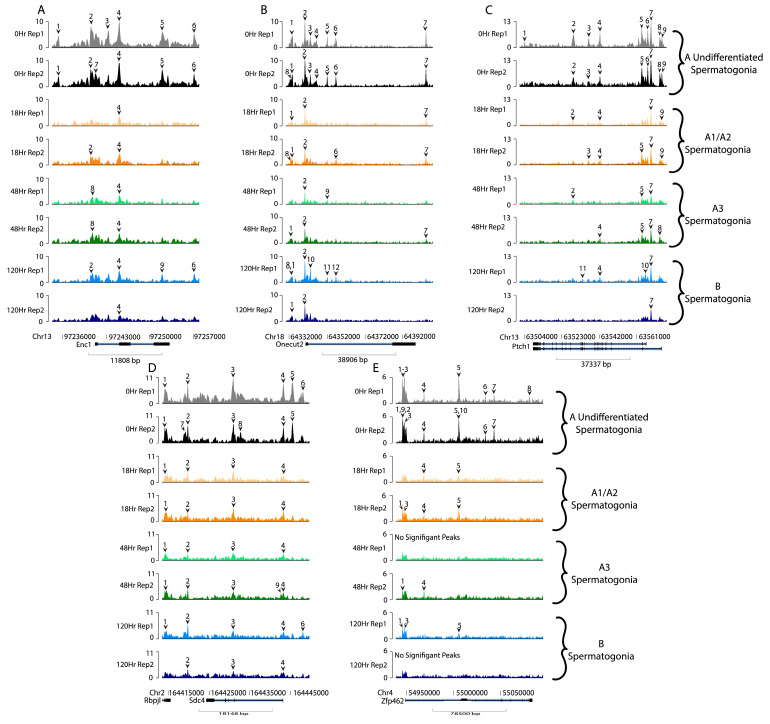
Accessibility of uncharacterized undifferentiated spermatogonia genes. Accessibility of uncharacterized genes, which are highly expressed at the 0 h timepoint but then decrease 18 h post-RA [5]. Numbered carats denote significant accessibility peaks for each gene. Relative sequencing tracks the mapping accessibility of these uncharacterized genes including (**A**) Enc1 (**B**) Onecut2 (**C**) Ptch1 (**D**) Sdc4, and (**E**) Zfp462. The timepoints and each of their replicates are represented in different rows and by color: 0 h (grey and black), 18 h (yellow and orange), 48 h (light and dark green), and 120 h (light and dark blue).

While global trends did not indicate a functional link between chromatin accessibility and transcription, we examined specific genes with known transcriptional patterns during spermatogonial development. We found that for genes that have decreased transcript abundance following RA action, including *Nanos2*, *Lin28a*, *Neurog3*, *Pou5f1*, *Sox3*, *Zbtb16*, *Enc1*, *Onecut2*, *Ptch1*, *Sdc4*, and *Zfp462*, there was accessible chromatin at all times, but these genes showed the most accessibility in undifferentiated spermatogonia where there were more peaks and/or a greater peak amplitude (Figure 5 and Figure 6). However, without further investigation, this remains only a correlation between downregulated DEGs and decreased chromatin accessibility following RA action. We cannot rule out the possibility that this is simply a result of global chromatin compaction following RA action rather than a specific functional link tied to transcription of these genes.

**Figure 7:**
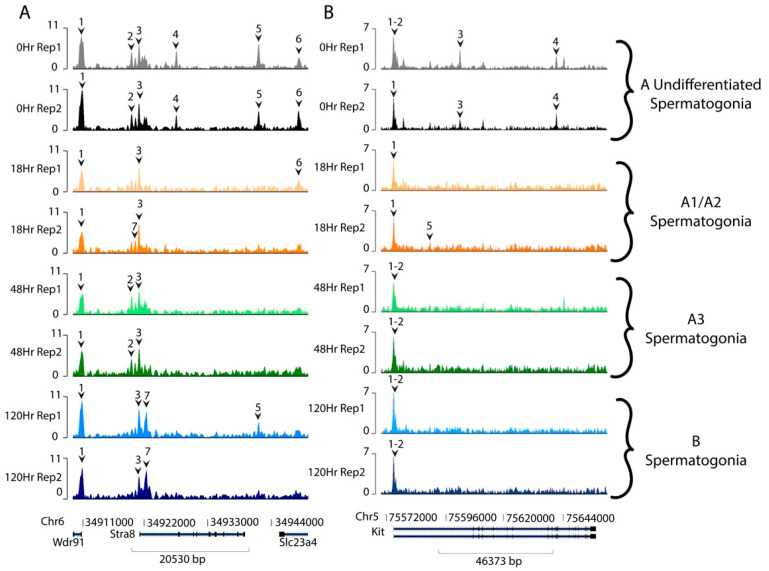
Accessibility of functional spermatogonia genes. Relative sequencing tracks mapping accessibility of common spermatogonial genes (**A**) STRA8 and (**B**) KIT, both of which show changes in peak amplitude and width between the undifferentiated and differentiating spermatogonia. Numbered carats denote significant accessibility peaks for each gene. The timepoints and each of their replicates are represented in different rows and by color: 0 h (grey and black), 18 h (yellow and orange), 48 h (light and dark green), and 120 h (light and dark blue).

Genes with increased expression, including biologically relevant markers of differentiating spermatogonia such as *Stra8* and *Kit*, were also accessible at all times (Figure 7). STRA8 has been shown to bind its own promoter and act as a transcriptional activator of many other genes in preleptotene spermatocytes at the onset of meiosis [41]. While it is unknown whether this transcriptional program activated by STRA8 has epigenetic underpinnings, our data suggest that STRA8 does not utilize an increase in chromatin accessibility at the time of RA action at the A-to-A1 transition. Additionally, previous investigations of *Kit* have suggested it may be epigenetically controlled at spermatogonial differentiation [43]. Our data indicate that, similar to the decrease in accessibility seen in undifferentiated spermatogonial markers following RA action, relevant markers of differentiating spermatogonia exhibited the same decrease in accessible chromatin at the A-to-A1 transition but the chromatin was never fully inaccessible. The data from known differentiated spermatogonial markers would instead indicate a global, rather than transcriptionally functional, occurrence of chromatin compaction.

We found that ~73% of genes are not only accessible over all four timepoints (Figure 2), but their accessibility predominantly occurred around the TSS (Figure S3). As a result of the fact that genes remain largely accessible at the TSS, other factors must bind to this region to control transcription of those genes. The identification of these other factors would be a more direct way to assess how the transcriptional program is triggered at the A-to-A1 transition. Some possibilities may be suggested from previous studies of epigenetic modifiers at other stages of spermatogenesis. For instance, high 5-hydroxymethycytosine (5hmC) levels and a greater abundance of RNA polymerase II have been shown to correlate with higher gene expression during meiosis [44,45,46]. Histone modifications such as H2K27me3 and H3K4me3 have also been examined in spermatogonial stem cells (SSCs) and suggest another epigenetic control mechanism that could possibly extend into spermatogonial development [47,48]. Further study of these modifications would be necessary to assess their influence in each of the spermatogonial subtypes.

Retinoic acid triggers complex changes at both the genetic and morphological levels. These data build on previous chromatin characterization in spermatogonia to show where in the genome chromatin compaction occurs. On a global scale, there does not appear to be a functional, predictive link between chromatin compaction and transcription in the examined spermatogonial populations. Future work on potential transcription factors acting in areas of open chromatin may help to further elucidate the complex network of transcriptional changes induced by RA.

## Data Availability

The data discussed in this publication have been deposited in NCBI’s Gene Expression Omnibus [49] and are accessible through GEO: series accession number GSE197484.

## Supplementary Materials

The following supporting information can be downloaded at: https://www.mdpi.com/article/10.3390/life13030690/s1, Figure S1: Reproducibility; Figure S2: Region proximity to gene bodies; Figure S3: Fragment enrichment over gene body; Figure S4: Peaks location and regulation during spermatogonial development; Figure S5: Transcript values of accessible genes; Figure S6: Gene ontology.
