# Supplementary material for: Chromatin Remodeling via Retinoic Acid Action during Murine Spermatogonial Development
Source: Life (Basel). 2023 Mar 3;13(3):690. doi: 10.3390/life13030690 (PMC10058303; doi:10.3390/life13030690)
Supplement: Supplementary file 1 [file life-13-00690-s001.zip › life-2244833-supplementary.pdf]

life

# Supplementary material of Chromatin Remodeling via Retinoic Acid Action During Murine Spermatogonial Development

**Christine Schleif [1], Rachel Gewiss [1] and Michael Griswold [1,*]**

[1] Center for Reproductive Biology, School of Molecular Biosciences, Washington State University, Pullman, 99164, DC, USA
* Correspondence: mgriswold@wsu.edu

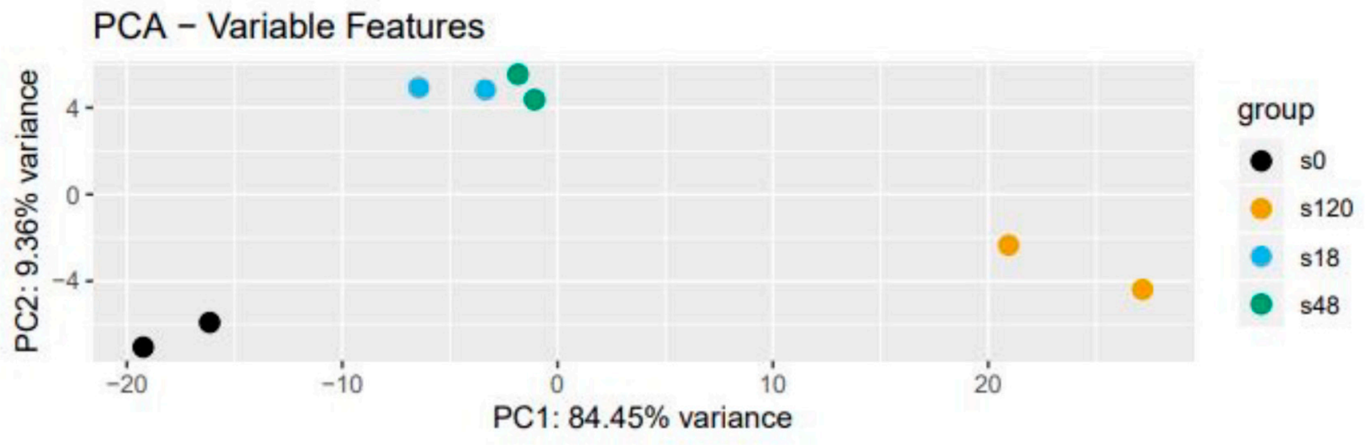


**Figure S1.** Reproducibility. PCA plot for each biological replicate and time point. Close clustering reveals good replicability between biological replicates. s0= 0 hour samples, s18= 18 hour samples, s48= 48 hour samples, s120= 120 hour samples.

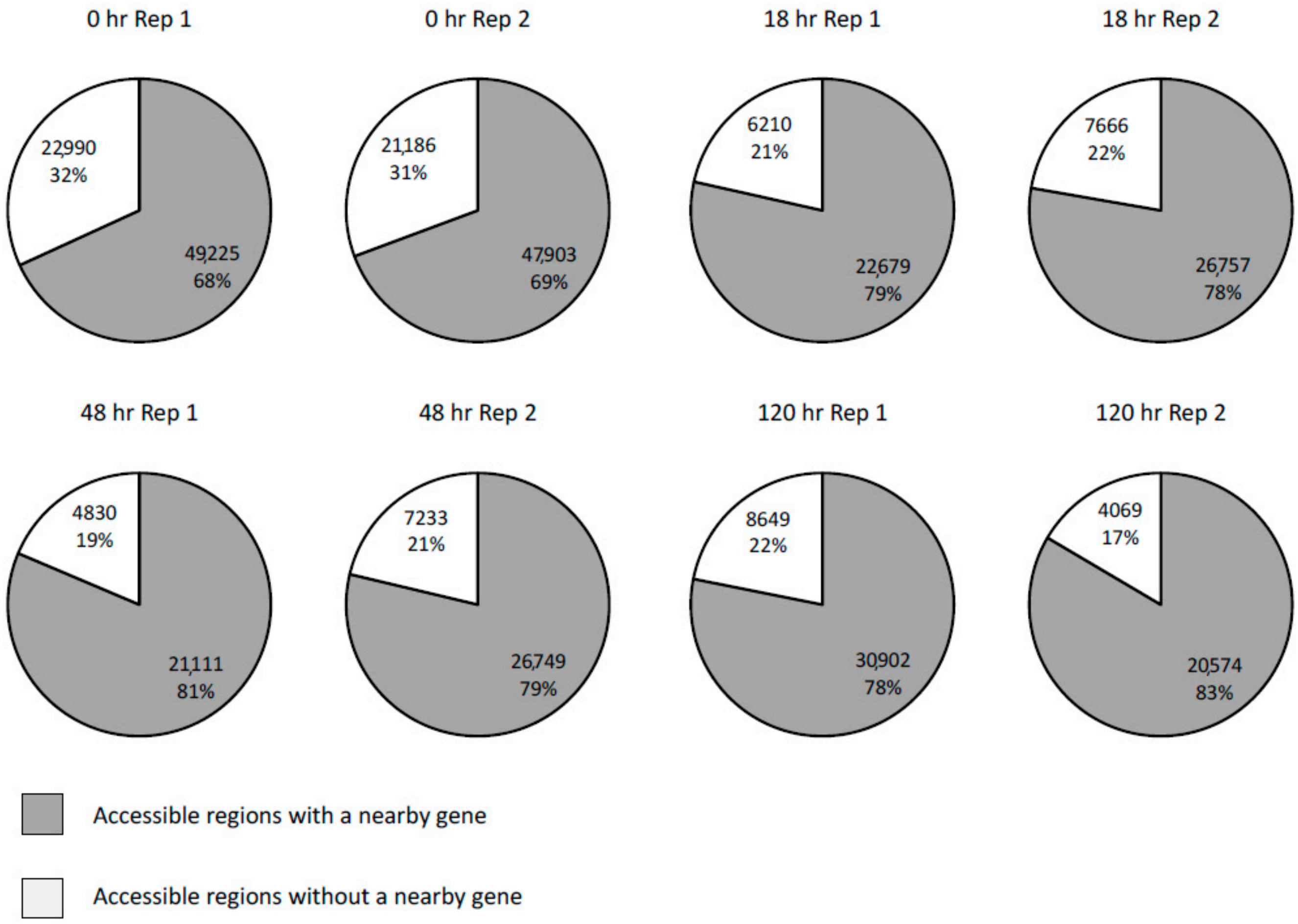


**Figure S2.** Region Proximity to Gene Bodies. Percent of accessible regions either with or without nearby genes for each time point and replicate. Gray sections show the accessible regions with a gene(s) nearby, white sections show the accessible regions that do not have a gene within 10 kb of the region.

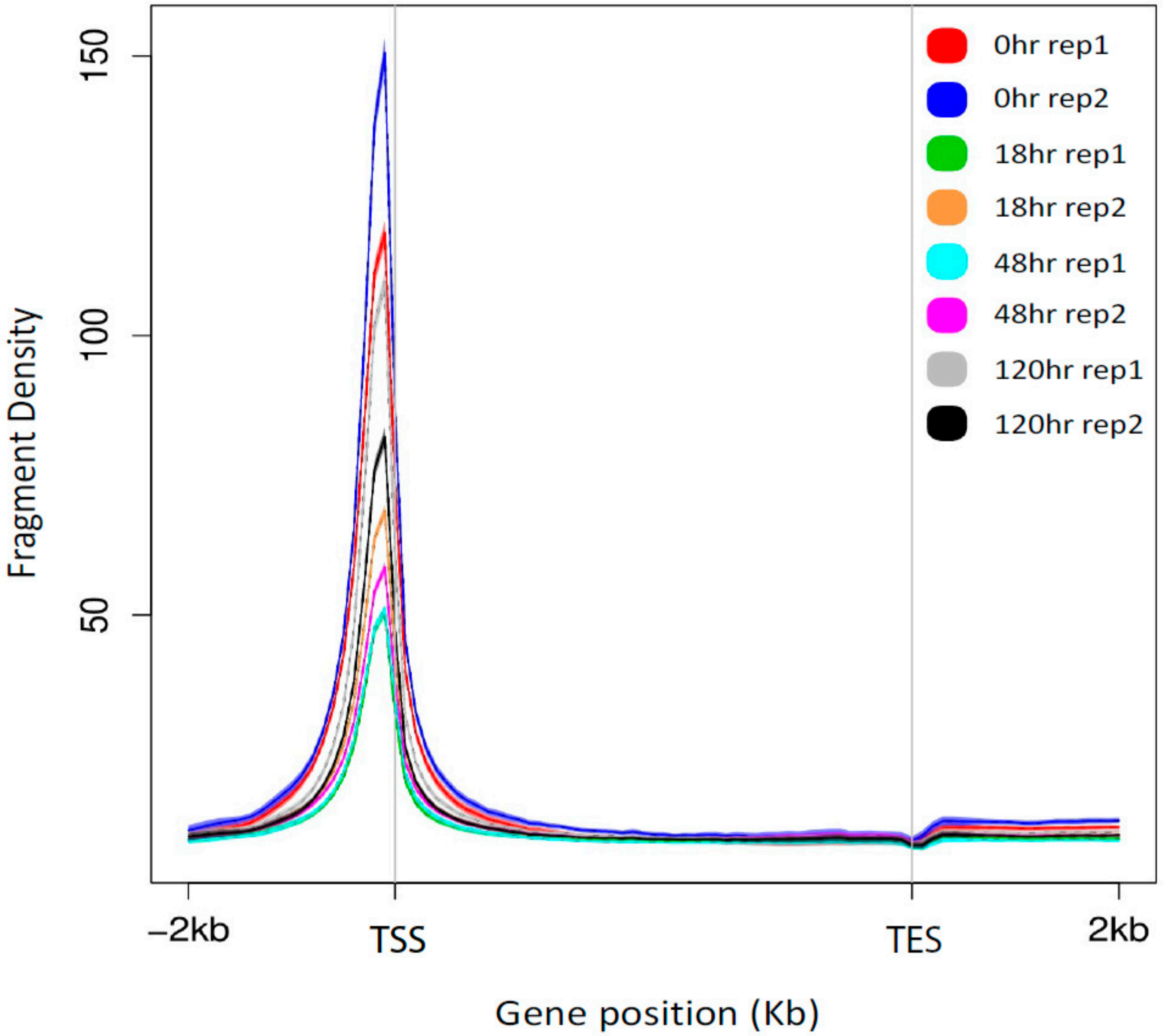


**Figure S3.** Fragment Enrichment Over Gene Body. Plotting peak enrichment over the gene body for all time points (0, 18, 48, and 120 hours post-RA) and biological replicates (rep1 or rep2) which showed the most accessibility occurs around the transcription start sites. Accessibility is noted here in terms of fragment density of transposition events along genes within the genome. Peaks at 0bp correspond with the either the transcription start sites (TSS) or transcription end site (TES) of any given gene in the genome.

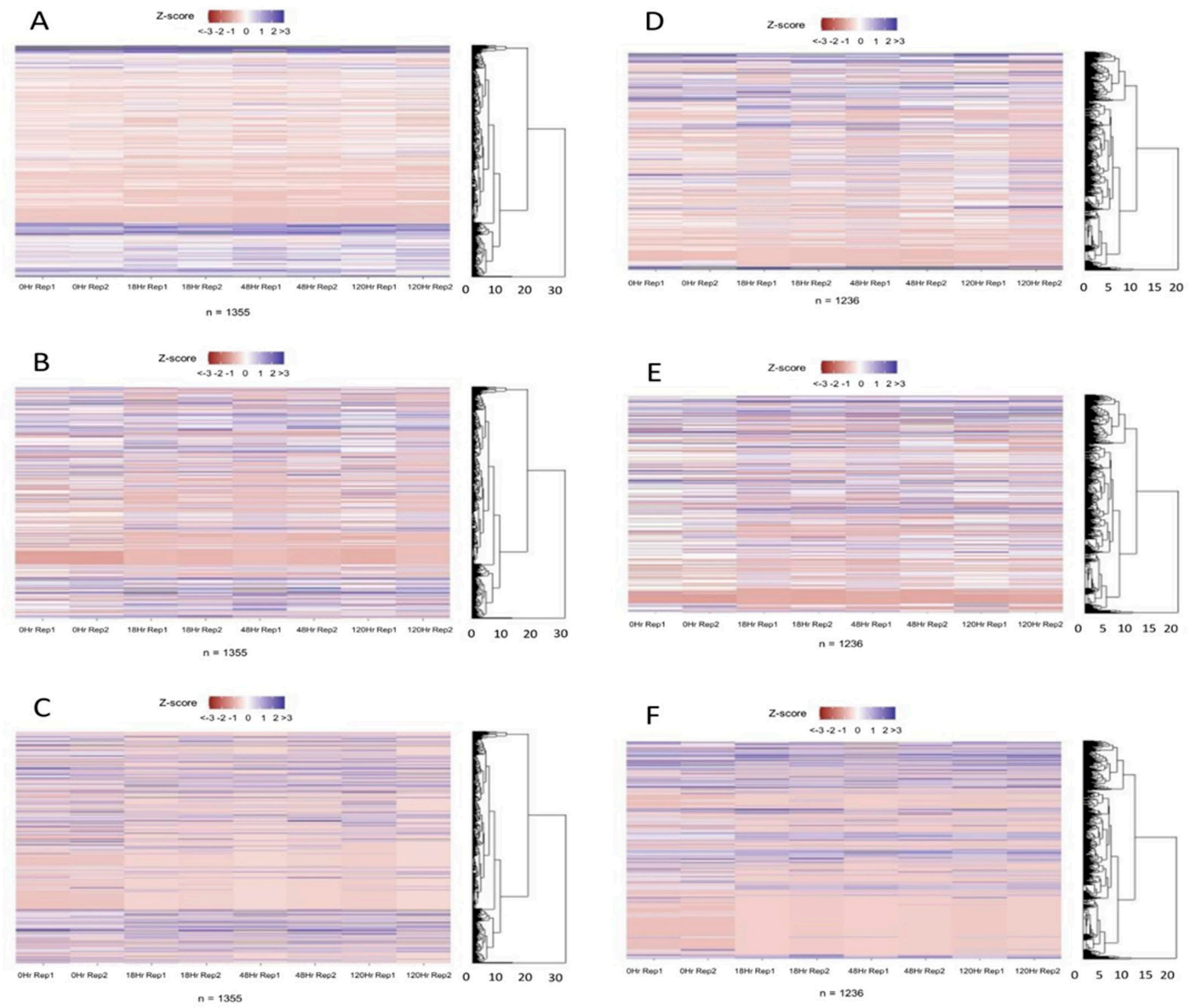


**Figure S4.** Peaks Location and Regulation During Spermatogonial Development. Heatmap of variance of peaks for genes downregulated (A, B, C) and upregulated (D, E, F) by 120 hours post-RA for peaks downstream of a gene subsetted by peaks within (A,D), upstream (B, E) or down stream (C. F) of their associated gene. Clustering reflects that of heatmaps showing all peakd for displayed genes (Figure 3). Numver of peaks and plotted Z-scores provided in Supplementay file.

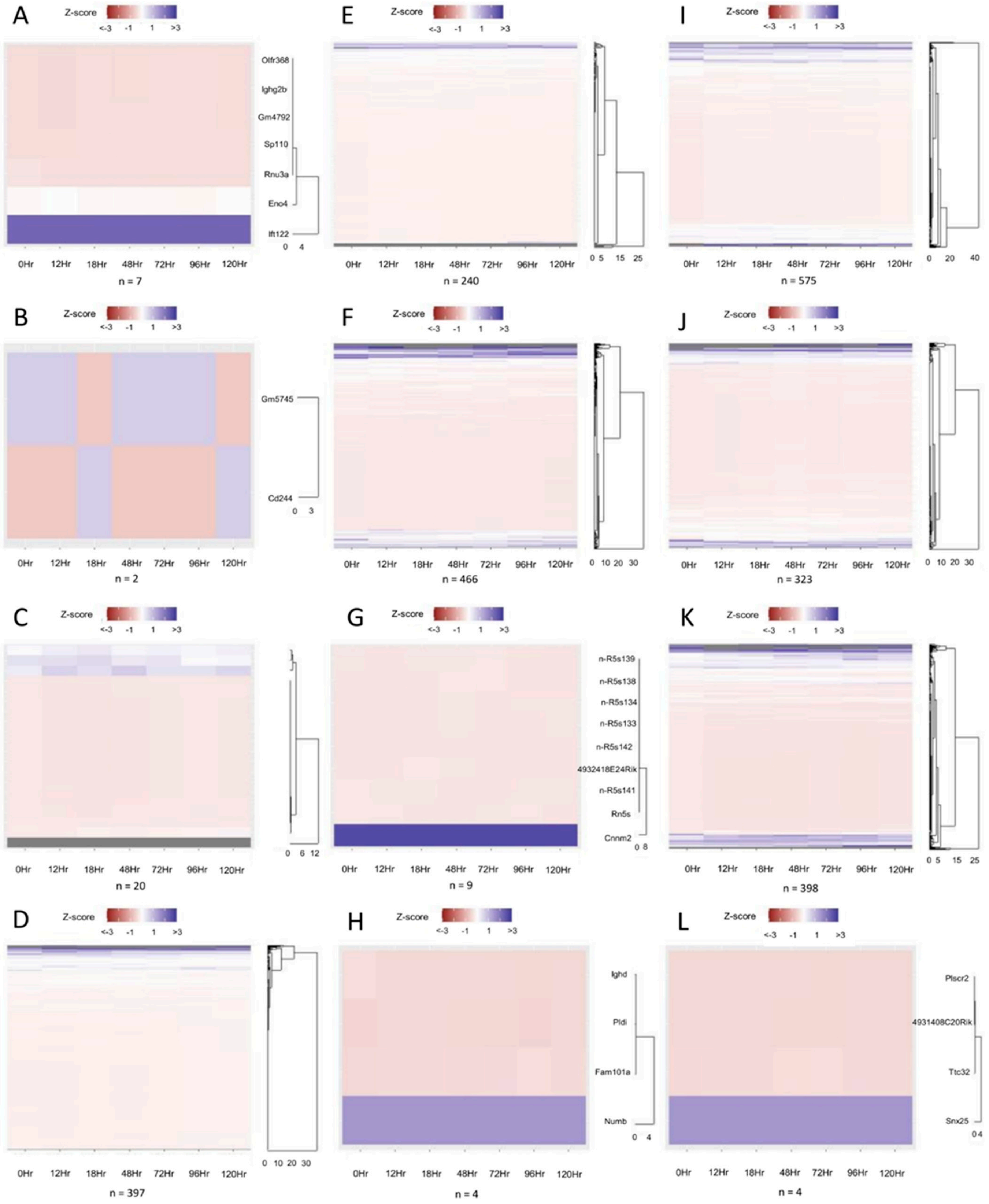


**Figure S5.** Transcript Values of Accessible Genes. Heatmaps of transcript values during spermatogonial developmetn for genes which have accessible chromatin at A) 18 hours only, B) 48 hours only, C) 120 hours only, D) 0 and 18 hours, E) 0 and 48 hours, F) 0 and 120 hours, G) 18 and 120 hours, H) 48 and 120 hours, I) 0, 18,and 48 hours, J) 0, 18 and 120 hours, K) 0, 48, and 120 hours and L) 18, 48, and 120 hours. There were no genes which were solely accessible at the combination of 18

and 48 hours. Transcript values and plotted Z-scores available in Supplimentary File. Transscript data from [5].

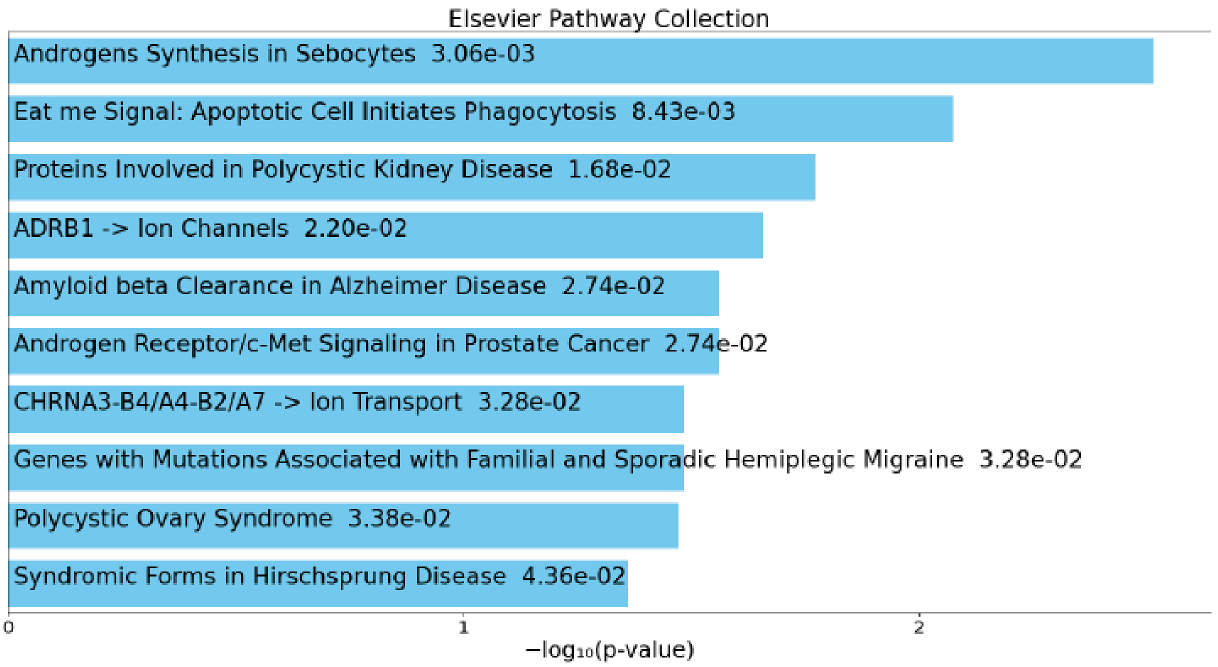


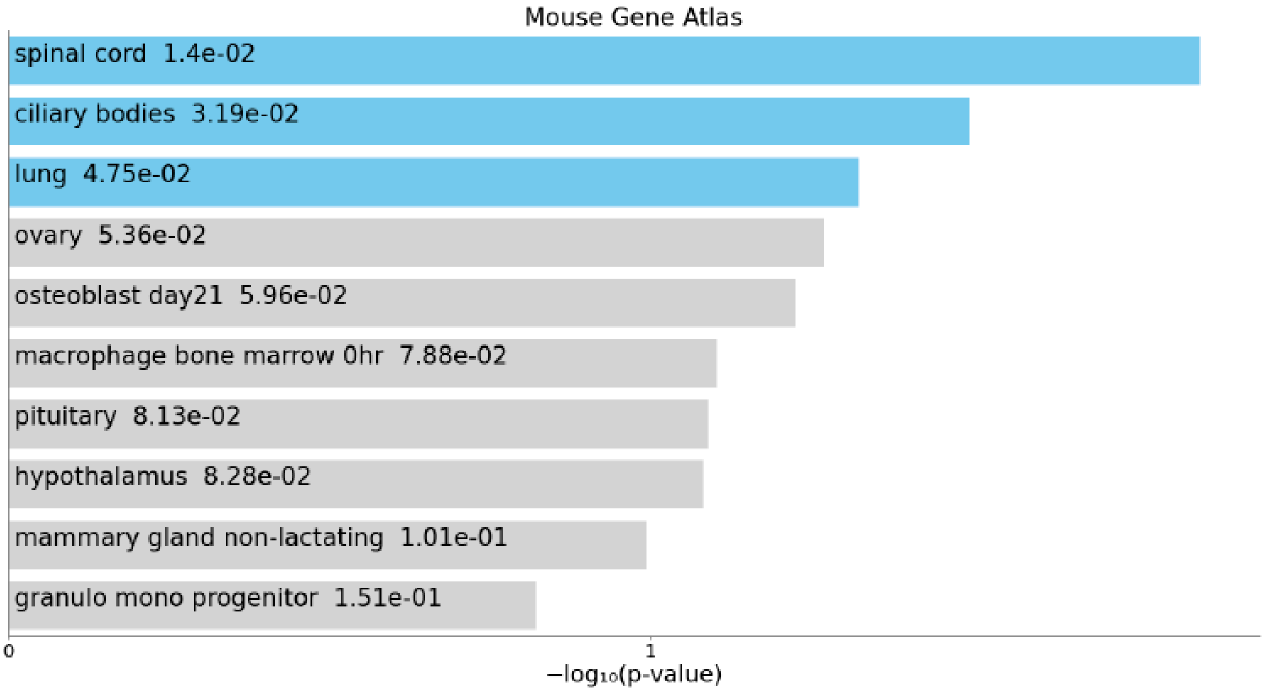


**Figure S6.** Gene Ontology. Genes with associated accessible chromatin only at 0 hours post-RA that also displayed decreased transcript levels following RA action at 12, 18, 48, 72, 96, and/or 120 hours post-RA. Testis and germ cell functions and cell types were not enriched among the dataset. Blue bars show terms with statistical significance, gray bars show top terms that are not statistically significant. p-values are shown within each bar.
